# ID 136 - Efficacy and Safety of Biologics Approved in Brazil for Moderate-Severe Crohn's Disease: a systematic review with network meta-analysis

**DOI:** 10.5327/2237-9622.2025.v34s1.136

**Published:** 2025-11-25

**Authors:** Daniela Gorski, Raul Edison Luna Lazo, Dalton de Assis de Souza, Helena Hiemisch Lobo Borba, Fernanda Stumpf Tonin, Roberto Pontarolo

**Keywords:** monoclonal antibody, remission induction, inflammatory bowel disease

## Abstract

**Introdução:**

Although immunosuppressants, aminosalicylates, and corticosteroids are initially prescribed to manage Crohn's disease (a chronic inflammatory bowel disorder) 45% of patients develop resistance to these first-line therapies, requiring second-line biologics1. The Brazilian Unified Health System (SUS) offers some biologics for patients’ remission induction: infliximab [5 mg/kg weekly intravenous (IV)], adalimumab [160 mg initially, 80 mg after one week, and 40 mg every two weeks subcutaneous (SC)], certolizumab pegol (400 mg weekly SC), vedolizumab (300 mg IV), and ustekinumab (6 mg/kg IV)2. We aimed to synthesize the evidence on the clinical effects of biological drugs approved in Brazil for remission induction in moderate-severe Crohn's disease in adults, regardless of primary indication.

**Método:**

A systematic review of randomized controlled trials was performed according to Cochrane Collaboration recommendations, with searches in PubMed, Scopus, Web of Science (February-2024). Data from the last week of follow-up for each outcome of interest [remission, serious adverse events (SAE)], were pooled and analyzed using network meta-analysis with surface under the cumulative rating curve analysis (SUCRA). Results were reported as risk ratios with 95% credibility intervals (R/RStudio).

**Resultados:**

Thirty trials (n=10,105) (1997-2022) assessing 12 biologics across 43 different regimens were included. Infliximab had the highest probability of inducing remission (SUCRA 96%). The remaining SUS-provided biologics for this disease presented low-to-intermediate efficacy (remission probabilities varying from 38-70%). Conversely, newer biologics as natalizumab [6mg/kg IV two infusions] and gulselkumab [200, 600 or 1200mg] had higher remission rates (SUCRA 75-84%). The adalimumab regimen of 160mg initially, 80mg after one week, and 60mg every two weeks outperformed the regimen of 40mg every two weeks (remission probabilities of 92% vs. 72%). Certolizumab was more prone to cause SAE (71%), followed by adalimumab (63%), vedolizumab (62%), natalizumab (60%), ustekinumab (56%). Guselkumab 1200mg was the safest alternative (SAE probability of 18%), with 200mg and 600mg doses also showing lower event rates (<50%).

**Conclusão:**

Natalizumab and guselkumab appear to be promising alternatives (favorable clinical risk-benefit ratio) for treating moderate-severe Crohn's disease. Currently, there are no specific recommendations from the Brazilian Clinical Protocol and Therapeutic Guidelines2 or reports from CONITEC (Comissão Nacional de Incorporação de Tecnologias no SUS) for using these biologics in remission induction. International guidelines already endorse natalizumab for both induction and maintenance of remission in moderate-severe cases3. Guselkumab, primarily approved for psoriasis in several countries, is under appraisal by international health technology agencies as the National Institute for Health and Care Excellence (NICE) for Crohn's disease, based on 48-week results from the Phase 3 GALAXI (NCT03466411) and GRAVITI (NCT05197049) trials4. Further assessments including drugs’ access and costs should be performed to ensure that these technologies provide added value for the Brazilian health system.

